# Testing the association between shoulder pain prevalence and occupational, physical activity, and mental health factors in two generations of Australian adults

**DOI:** 10.1186/s12998-023-00520-1

**Published:** 2023-11-27

**Authors:** Christopher J. Hodgetts, Angela Jacques, Lee Daffin, Yvonne C. Learmonth

**Affiliations:** 1 Discipline of Chiropractic, Murdoch University, 90 South Street, Murdoch, WA 6150 Australia; 2 Discipline of Exercise Science, Murdoch University, 90 South Street, Murdoch, WA 6150 Australia; 3 Centre for Molecular Medicine and Innovative Therapeutics, Health Futures Institute, Murdoch University, Perth, WA Australia; 4 Centre for Healthy Ageing, Health Futures Institute, Murdoch University, Perth, WA Australia; 5 Institute for Health Research, University of Notre Dame Australia, Fremantle, WA Australia; 6 Perron Institute for Neurological and Translational Science, Perth, WA Australia

**Keywords:** Shoulder pain, Epidemiology, Prevalence, Occupation, Occupational factors, Physical activity, Depression, Anxiety

## Abstract

**Background:**

Shoulder pain is common among the adult population, but it appears to reduce in prevalence around retirement age. Associations between shoulder pain and work-place exposures, physical activity, or mental health status are unclear and may change with age. This study aimed to determine the prevalence of self-reported shoulder pain in Australian adults across two generations and test the association with occupational factors, physical activity, and mental health.

**Methods:**

In this cross-sectional study we used data from a longitudinal Australian pregnancy cohort (the Raine Study). We analysed data from the children (Gen2) at the 22-year follow-up (N = 1128) and parents (Gen1) at the 26-year follow-up (N = 1098). Data were collected on self-reported shoulder pain, occupational factors (employment status and work description), physical activity, and mental health at the respective follow-ups. Prevalence rates were provided as percentages with 95% confidence intervals. Univariate analysis for group comparisons included chi squared for categorical comparisons. The association of predictor variables and shoulder pain was assessed using logistical regression.

**Results:**

In Gen1 31.4% of adults aged 40–80 reported the presence of shoulder pain in the last month, with no significant difference between females and males. Gen1 participants younger than 65 reported more shoulder pain (OR[95%CI] = 1.80 [1.04–3.09]). Gen2 females (14.7%) reported shoulder pain in either shoulder more frequently than males (7.7%) and bilateral shoulder pain (8.0%) more frequently than males (1.9%). Gen1 had increased odds of reporting shoulder pain if their work was “physical or heavy manual” compared to “sedentary” (OR [95% CI] = 1.659 [1.185–2.323]) and when categorised with depression (OR [95% CI] = 1.940 [1.386–2.715]) or anxiety (OR [95% CI] = 1.977 [1.368–2.857]). Gen2 participants with depression (OR [95% CI] = 2.356 [1.620–3.427]) or anxiety (OR [95% CI] = 2.003 [1.359–2.952]) reported more shoulder pain.

**Conclusion:**

Overall, shoulder pain was more prevalent in young females than males and was more prevalent in those under the age of 65. Cross-sectional associations were established between some occupational factors in older adults and depression in all adults, and shoulder pain.

## Highlights/clinical messages

In the younger generation females had significantly higher rates of shoulder pain and in particular were almost four times as likely to report bilateral shoulder pain.

In the older generation, those over the ages of 65 were less likely to report shoulder pain.

In the older generation, physical or heavy work is associated with higher rates of shoulder pain.

In both generations depression and anxiety were associated with higher rates of shoulder pain.

Physical activity was not associated with shoulder pain in either generation.

## Background

Shoulder pain is common among the adult population, but estimates have wide ranges for point (7–26%) and one-month prevalence (17–31%) [1]. Although there are limited studies on the economic burden of shoulder pain, the cost is likely sizable with 250,000 rotator cuff repairs performed annually in the United States costing close to 3 billion USD [2]. Shoulder disorders lead to lost time off work and are associated with poorer general health and poor mental health as evidenced by increased levels of depression, anxiety and disturbed sleep [3–6].

It is unclear whether mechanical workplace exposures (such as repetitive movements, postures, or heavy loads) and levels of physical activity may predispose individuals to develop shoulder pain or specific disorders. Two recent systematic reviews concluded that there is limited-moderate evidence to suggest occupations requiring arm elevation and shoulder load are associated with shoulder disorders [7, 8]. The Australian Institute of Health and Welfare (AIHW) investigated occupational-related shoulder injuries presenting to general practitioners (GP) and determined 13% were classified as “work-related” [9]. Occupations that have a higher incidence of reported rotator cuff disorders are heavy labourers, those workers whose jobs required repetitive positioning in horizontal or above such as painters, and overhead athletes (e.g. swimming and throwing sports) [10, 11].

Currently younger populations dominate the data concerning the association between occupational factors and shoulder pain reporting. However, a 2015 systematic review demonstrated that despite rotator cuff degeneration increasing linearly with age, shoulder pain peaks between 55 and 64 and does not continue in the same linear fashion past the common retirement age of 65 [12]. While retirement itself may be responsible for the reduction in estimates, several studies have also shown a reduction of musculoskeletal pain reporting in older adults [13, 14].

Psychological health status appears to be related to shoulder pain and disability. In a cross-sectional analysis depression and anxiety were correlated with shoulder pain intensity [15]. It has also been reported that patients with shoulder disorders demonstrated a high prevalence of depression, anxiety and sleep disturbance when compared to a healthy control group [6]. A population based longitudinal study demonstrated a bidirectional relationship between pain and depression [16]. To this point there have been no longitudinal studies to suggest a direction for this relationship to shoulder pain.

Occupational, physical activity and psychosocial exposures may change during and after retirement age resulting in a reduction of shoulder complaints. Our aim is to determine the prevalence of shoulder pain in Australian adults and test the association with occupational factors, physical activity and mental health. In order to do this we will:

1. Establish the point-prevalence estimates of shoulder pain in the young, and one-month prevalence estimates of the older adult population in Australia.
2. Determine if occupational factors are associated with shoulder pain in young and older adult populations.
3. Determine if levels of activity are associated with shoulder pain in young and older adult populations.
4. Determine if depression or anxiety is associated with shoulder pain in young and older adult populations.

## Methods

### Study design, setting and participants

A cross-sectional analysis to assess the association between occupational, physical activity, mental health factors and self-reported shoulder pain. Data were accessed from a Western Australian pregnancy cohort study called the Raine Study [17–19]. The primary study recruited 2868 mothers between May 1989 and November 1991 from the largest maternity hospital in Perth, Western Australia (King Edward Memorial Hospital).

Following birth, the children (Generation 2- Gen2) of these women were serially followed through to adulthood (27 years of age), with data points at years 1, 2, 3, 5, 8, 10, 13, 16, 20, 23 and 27. Of the 2262 eligible children, 1134 completed the Gen2-22 year follow-up between 2012 and 2014 at the University of Western Australia Centre for Sleep Science. The mothers and fathers have been labelled Generation 1 (Gen1) and had provided limited data at pre-natal and peri-natal time points as well as every follow-up Gen2 was involved with. At the Gen1-26 year follow-up the participants completed their first exclusive follow-up, where much more extensive data was provided.

All aspects of the Raine Study have been approved by the Human Ethics Commitees at King Edward Memorial Hospital, Princess Margaret Hospital, University of Westen Australia and Curtin Univeristy. This cross-sectional analysis study, analysing musculoskeletal data, was reviewed and received approval from the Murdoch University Human Research Ethics Committee (project number: 2019/238).

### Data collection

The participants underwent a clinical assessment and a questionaire that included information about occupation, physical activity, mental health and musculoskeletal pain. The parents (Gen1) were invited to participate in the Raine Study Parent Assessment from 2016 to 2018, with 1098 completing the main questionnaire. The questionnaire included information about occupation, physical activity, mental health and musculoskeletal pain, as described in Tables 1 and 2.

### Demographic variables

Participants’ age (in years), sex (female or male) and smoking status (yes/no) were collected via the participant questionnaire. Age groups were defined based on a retirement age of 65 for Gen1. Participants heights were measured with a Holtain Stadiometer and body weight with a Wedderburn Chair Scale. BMI was calculated using the standard equation of BMI = weight (kg)/height (m)^2^.

**Table 1:** Shoulder pain outcomes

	Gen1	Gen2
Shoulder pain	Reporting of pain for Gen1 participants was from a one-month time period. Shoulder pain data were collected for participants via the Orebro Musculoskeletal Pain Questionaire (OMPQ) at the Gen1-26year follow-up. Participants were asked “ Please indicate the sites below in which you have had pain in the last month”, with options including left shoulder or right shoulder.	Reporting of pain for Gen2 participants was from a single point in time (point prevalence). Shoulder pain data were collected for participants via musculoskeletal pain questions within the Gen2-23 year follow-up questionnaire. Participants were asked “Do you currently have any body pain?”. For positive responses, the follow-up question asked “Where do you have pain?”, with options including left shoulder or right shoulder.

Shoulder pain outcomes were recoded to produce “any shoulder pain”, “unilateral shoulder pain”, and “bilateral shoulder pain” for both Gen1 and Gen2

**Table 2:** Potential predictor variables

	Gen1	Gen2
Occupational Factors		
Employment Status	Participants were asked to answer the following question: “Which of the following best describes your current employment situation?”. Options were: employed full-time; employed part-time; employed, but away from work (e.g. long service leave); unemployed looking for full time work; unemployed looking for part time work; not in the labour force (retired, not looking for work, unable to work); do paid casual work; doing unpaid or voluntary work; other.	Participants were asked to answer the following question: “What are you doing now?”. Options were: studying full-time; studying part-time; an apprenticeship; working full-time; working part-time; looking for work; carer for my child; carer for a family member; other. Participants were then asked “do you currently have a full-time, part-time or casual job of any kind?”. The options were: no, do not have a job – not seeking work; no, do not havea job – actively seeking work; yes, do work for payment or profit; yes, do unpaid work in a family business; yes, do other unpaid work.
Length of time in current occupation	For those participants that had reported they were currently working, they were asked to report how many years or months they had been working in their current occupation or job.	
Industry code	Participants were asked to report what industry do they work in for their current job. They were provided a list of industry codes.	
Work hours	Participants were asked to report how many hours per week they usually work in all (current) jobs: 1–15; 16–24; 25–34; 35–39; 40; 41–48; 49–55; more than 55. Those participants that reported being unemployed or retired where asked to list the main jobs that had in the past 5 years, the industry code and approximate years and months in that role.	Participants were asked to report how many hours per week they usually work in the last 7 days. Those participants that reported being unemployed or retired where asked to list the main jobs that had in the past 5 years, the industry code and approximate years and months in that role.
Description of work	Participants were asked to indicate on a scale of 1 (not al all) to 10 (extremely) if their work was “heavy or monotonous”. Participants were also asked to select which statement best described the work they do for their current job: sedentary occupation (e.g. secretary – where you spend most of your time sitting); standing occupation (e.g. shop assistant, security guard, spend most of your time standing/walking but not intense physical effort; Physical work (e.g. plumber, nurse – a job that requires some physical effort incuding handling of heavy objects and use of tools); heavy manual work (e.g. bricklayer – a job that involves very vigorous physical activity including handling very heavy objects).	
Physical activity	International Physical Activity Questionnaire (IPAQ). The participants were asked to report the number of days and hours of vigorous, moderate and walking based physical activity in the last 7 days. Using these measures, the participants were classified as either low, moderate or vigorous levels of exercise. They were also asked to report how many hours they spent sitting on weekdays and weekends in the past 7 days.	
Depression and anxiety	Depression Anxiety Stress Scale (DASS21) [20]. Participants were asked to respond to 21 statements on a 0–3 scale. The sub scale scores were used to classify the patient as having normal, mild, moderate or severe symptoms of depression. We dichotomized this variable into ‘no depression’ symptoms and ‘depression’ symptoms. We followed the same process for the anxiety subscale.	

### Statistical methods

Descriptive statistics of sample demographic data were based on means and standard deviations for normally distributed continuous data or medians and interquartile ranges for non-normally distributed continuous data. Prevalence rates were provided as percentages with 95% confidence intervals.

Within group univariate categorical comparisons were done using chi squared tests. The association of demographic predictor variables with shoulder pain was assessed using logistic regression models including interaction with age and sex for Gen 1 and Gen 2 respectively. Results were summarized using odds ratios (OR) and 95% confidence intervals (CI). Data were analysed using IBM SPSS statistics for Mac (version 24; IBM Corp., Armonk, NY).

## Results

### The sample

There was shoulder pain and demographic data for 1098 participants in Gen1 and 1128 in Gen2. Gen1 participants ranged from 40 to 80 years of age, with 57.9% being female. Gen2 participants were between 20 and 24 years of age, with 52.9% of participants being female. Table 3 provides descriptive data (demographic, clinical and social) for the two generation samples. Data are presented as mean and standard deviations or number (%) unless otherwise stated. This provides an overview of the two sample populations.

**Table 3:** Descriptive statistics, employment status and occupational status for Gen1 and Gen2 samples

	Gen1 (n = 1098) Mean (SD) or N (%)	Gen2 (n = 1128) Mean (SD) or N (%)
**Age (y)**	56.55 (5.73)	22.2 (0.64)
**Sex (f)**	636 (57.9%)	597 (52.9%)
**Weight (kg)**	79.35 (23.88)†	72.4 (21.9)†
**Height (m)**	1.68 (0.14)†	1.72 (0.14)†
**BMI**	27.34 (6.83)†	23.9 (5.45)†
**Waist-Hip Ratio**	0.91 (0.08)	0.83 (0.07)
**Smoking (yes)**	498 (45.4%)	185 (16.4%)
**Employment Status**		
Full Time Part Time Retired Other* Unemployed Casual Work Voluntary Work or Carer Not Reported	489 (44.5%) 244 (22.2%) 148 (13.5%) 60 (5.4%) 48 (4.4%) 43 (3.9%) 18 (1.6%) 48 (4.4%)	442 (39.2%) 303 (26.9%) NR 50 (4.4%) 154 (13.7%) NR 34 (3.0%) 145 (12.8%)
**Study Status**		
Full-time Part-time Apprentice Not studying	NA NA NA NA	377 (33.4%) 99 (8.8%) 29 (2.6%) 623 (55.2%)
**Occupational Status**		
Sedentary Standing Physical Work Heavy Manual Work Not Reported	432 (39.3%) 188 (17.1%) 194 (17.7%) 22 (2%) 262 (23.9)	273 (24.2%) 332 (29.4%) 283 (25.1%) 71 (6.3%) 169 (15%)

*N*, number; *SD*, standard deviation; *†*, Median (inter-quartile range); *, other includes being on long service leave, self-employed, business owner/proprietor, musician, artist, or house person; *NA*, not available

### Objective 1: prevalence rates

The respective prevalence rates of any, unilateral and bilateral shoulder pain were stratified by sex for both generations in Table 4 and by age for Gen1 in Table 5. There were no differences between male and female participants in Gen1 or for unilateral pain in Gen2, but there were higher rates of bilateral or any shoulder pain for females in Gen2. Gen1 participants that were younger than 65 had significantly higher odds of reporting shoulder pain (OR[95%CI] = 1.80 [1.04–3.09], p = 0.034). Gen2 participants that were female had significantly higher odds of reporting shoulder pain (OR[95%CI] = 2.07 [1.40–3.05], p < 0.001).

**Table 4:** Sample prevalence rates for Gen1 and Gen2 for male and female participants, %, 95% CIs

Prevalence period (shoulder pain type)	sample %	95% CI	Male %	95% CI	Female %	95% CI
**Gen1**						
SP 1-month	34.2	31.3–37.0	31.8	27.5–36.2	35.8	32.0-39.7
SP 1-month (UL)	21.8	19.2–24.1	19.9	16.1–23.7	23.1	19.6–26.4
SP 1-month (BL)	12.4	10.2–14.4	11.9	9.3–14.9	12.7	10.3–15.5
**Gen2**						
SP point	11.4	9.8–13.3	7.7*	5.4–10.0	14.7*	11.0-17.8
SP point (UL)	6.3	4.9–7.7	5.8	3.9–7.9	6.7	4.8–8.6
SP point (BL)	5.1	3.9–6.5	1.9*	0.9–3.1	8.0*	5.9–10.5

*CI*, Confidence interval; *SP*, shoulder pain; UL, unilateral pain; BL, bilateral pain; *, statistically significant difference between groups

**Table 5:** Sample prevalence rates for Gen1 stratified by five year age categories, 95% CIs

Age	N	sample %	95% CI
< 50 (N = 143)	50	35.0%	27.2–43.4
50–54 (N = 275)	104	37.8%	32.0–44.0
55–59 (N = 380)	125	32.9%	28.2–37.9
60–64 (N = 222)	78	35.1%	28.8–41.4
>=65 (N = 78)	18	23.1%	14.1–33.3

CI, Confidence interval; *N*, number

### Objectives 2–4: associations

#### Gen1

Table 6 displays the results of univariate analysis for Gen1, including prevalence rates cross tabulated with predictor variables and odds ratios with age interaction for each variable.

Objective 2: Participants in Gen1 had higher odds of shoulder pain if their occupations involved physical or heavy manual work as opposed to sedentary work (p = 0.003, overall interaction effect of age and 3 category work type: p = 0.005) or they worked in occupations with a higher perceived heaviness or monotony rating (p = 0.002). Employment status was not associated with shoulder pain. Including age as an interaction with employment status had no signficant effect (p = 0.800) as the majority of participants (95.3%) undertaking work were in the younger age group. Similarly, 98.0% of participants undertaking physical or heavy work were < 65.

Objective 3: There were no associations found between physical activity levels and shoulder pain. Including age as an interaction had no signficant impact on the physical activity levels odds ratios (interaction effect p = 0.775).

Objective 4: Gen1 participants had significantly higher odds (p < 0.001) of reporting shoulder pain when categorized with depression or anxiety. As the majority of participants with depression (95.8%) or anxiety (95.5%) were under 65, including age as an interaction had minimal impact on univariate depression or anxiety odds ratios.

**Table 6:** Univariate analysis for Gen1, N (%)

Variable	N	Prevalence	p-value	OR (95% CI)	p-value
Employment status Unemployed (ref) Employed	1050 196 854	73 (37.2%) 302 (35.4%)	0.620	1.00 0.92 (0.67–1.27)	0.620
Work description Sedentary (ref) Standing Physical or Heavy	836 432 188 216	137 (31.7%) 59 (31.4%) 94 (43.5%)	0.007	1.00 0.99 (0.68–1.42) 1.66 (1.19–2.32)	0.935 0.003
Work description*Age					
< 65					
Sedentary (ref) Standing Physical or Heavy	409 179 209	134 (32.8%) 55 (30.7%) 92 (44.0%)	0.008	1.00 0.91 (0.62–1.33) 1.61 (1.15–2.27)	0.627 0.006
Rating of heaviness ≤ 3/10 (ref) > 3/10	840 473 367	143 (30.2%) 149 (40.6%)	0.002	1.00 1.58 (1.19–2.10)	0.002
Rating of heaviness*Age					
< 65					
≤3/10 (ref) > 3/10	439 361	136 (31.0%) 147 (40.7%)	0.004	1.00 1.53 (1.14–2.05)	0.004
IPAQ Low (ref) Moderate High	1043 280 356 407	104 (37.1%) 126 (35.4%) 145 (35.6%)	0.887	1.00 0.93 (0.67–1.28) 0.94 (0.68–1.29)	0.649 0.685
Depression No Depression (ref) Depression	1019 853 166	281 (32.9%) 81 (48.8%)	< 0.001	1.00 1.94 (1.39–2.72)	< 0.001
Depression*Age < 65 No Depression (ref) Depression ≥ 65 No Depression (ref) Depression	788 159 65 7	267 (33.9%) 77 (48.4%) 14 (21.5%) 4 (57.1%)	< 0.001 0.039	1.00 1.83 (1.30–2.59) 1.00 4.86 (0.97–24.30)	< 0.001 0.054
Anxiety No Anxiety (ref) Anxiety	1025 893 132	300 (33.6%) 66 (50%)	< 0.001	1.00 1.98 (1.37–2.86)	< 0.001
Anxiety*Age < 65 No Anxiety (ref) Anxiety ≥ 65 No Anxiety (ref) Anxiety	826 126 67 6	287 (34.7%) 62 (49.2%) 13 (19.4%) 4 (66.7%)	0.002 0.009	1.00 1.82 (1.25–2.65) 1.00 8.31 (1.37–50.37)	0.002 0.021

*OR*, Odds Ratio; *CI*, Confidence interval; *N*, number

#### Gen2

Table 7 displays the results of univariate for Gen2, including prevalence rates cross tabulated with predictor variables and odds ratios with sex interaction for each variable.

Objective 2: Overall, employment status, work description or rated heaviness were not associated with shoulder pain. However, examination of gender subgroups showed that females who were employed; worked physical/heavy jobs (overall interaction for both p = 0.001); and rated their work as heavy had increased odds of reporting shoulder pain.

Objective 3: There was no association found between physical activity levels and shoulder pain reporting.

Objective 4: Gen2 participants with depression or anxiety had increased odds of reporting shoulder pain (p < 0.001). Including sex had minimal impact on univariate depression or anxiety odds ratios. Females with depression or anxiety had slightly higher odds of reporting shoulder pain compared to males, but these differences were not clinically meaningful.

**Table 7:** Univariate analysis for Gen2, N (%)

Variable	N	Prevalence	p-value	OR (95% CI)	p-value
Employment status Unemployed (ref) Employed	1128 196 932	29 (14.8%) 100 (10.7%)	0.104	1.00 0.69 (0.44–1.08)	0.106
Work description Sedentary (ref) Standing Physical or Heavy	959 273 332 354	27 (9.9%%) 36 (10.8%) 37 (10.5%)	0.930	1.00 0.94 (0.56–1.59) 1.04 (0.64–1.69)	0.818 0.868
Rating of heaviness ≤ 3/10 (ref) > 3/10	935 352 583	34 (9.7%) 68 (11.7%)	0.341	1.00 1.24 (0.80–1.91)	0.341
IPAQ Low (ref) Moderate High	1043 280 356 407	30 (14.0%) 27 (11.5%) 72 (10.6%)	0.408	1.00 0.80 (0.46–1.40) 0.73 (0.46–1.16)	0.433 0.182
Depression No Depression (ref) Depression	1075 760 315	69 (9.1%) 60 (19.0%)	< 0.001	1.00 2.36 (1.62–3.43)	< 0.001
Depression*Sex Male No Depression (ref) Depression Female No Depression (ref) Depression	382 112 378 203	26 (6.8%) 15 (13.4%) 43 (11.4%) 45 (22.2%)	0.026 < 0.001	1.00 2.12 (1.08–4.15) 1.00 2.22 (1.40–3.51)	0.029 < 0.001
Anxiety No Anxiety (ref) Anxiety	1075 811 264	81 (10.0%) 48 (18.2%)	< 0.001	1.00 2.00 (1.36–2.95)	< 0.001
Anxiety*Sex Male No Anxiety (ref) Anxiety Female No Anxiety (ref) Anxiety	402 92 409 172	30 (7.5%) 11 (12.0%) 51 (12.5%) 37 (21.5%)	0.159 0.006	1.00 1.68 (0.81–3.50) 1.00 1.92 (1.21–3.07)	0.163 0.006

*OR*, Odds Ratio; *CI*, Confidence interva*l; N*, number

## Discussion

The aim of this research was to determine the prevalence of shoulder pain and its association with three key domains: occupational factors, physical activity and mental health. In the Gen2 sample population almost twice as many females reported pain compared to males and more than four times as many females reported bilateral shoulder pain as their male counterparts. Around a third of adults aged 40–80 reported the presence of shoulder pain in the last month, with no significant difference between females and males. Within the older cohort there were higher rates of self-reported shoulder pain in those that reported their occupation involved physical or heavy manual work. This same association was not demonstrated in the younger cohort. Both samples had associations between depression or anxiety and shoulder pain reporting, but neither population had associations between physical activity level and shoulder pain.

The reporting of shoulder pain within the older adult generation was highest between the ages of 45 and 65, though these results cannot be considered significant as the confidence intervals overlapped. However, within Gen1 those over 65 were less likely to report shoulder pain than those under. These results are in line with research suggesting that shoulder pain prevalence steadily increases with age until 65 and then either remains stable or slightly decreases [12]. According to previous research those people still working past the age of 65 continue to report shoulder pain [21]. Therefore, the likely mechanism for this result is retirement and subsequent cessation of exposure to occupational factors that either cause or aggrevate shoulder pain.

Recent research has indicated that glenohumeral joint hypermobility (GJH) is an observable finding in female athletes across several varied sporting disciplines both recreational and elite. Features commonly associated with GJH include localised pain, ligamentous sprains, dislocations, and subluxations. Female athletes with GJH appear to have a threefold increase in shoulder concerns comparison to athletes without GJH [22]. Although a direct causal relationship is not inferred with respect to GJH and the current studies populations. Glenohumeral joint hypermobility may be a potential contributing factor that could account for the significant difference in reported shoulder pain between females and males in the younger generation in this study. Additionally, findings indicate that normalised general joint laxity in growing children (9 to 15 yrs) is greater in girls than boys and increases with age [23]. Findings of this nature may contribute to the reported difference in bilateral shoulder pain observed within the younger female population.

Our study investigated the relationship between shoulder pain and physical occupational factors in both generations. When considering shoulder pain and physical occupational factors the exposure-response relationship is not well understood [24]. However, several key occupational shoulder pain risk factors have been identified including repetitive motion, heavy loading, forceful actions, vibrational tasks and working with elevated arms [24–26]. The finding of this study suggest occupation may play a role in the development of shoulder pain. Research indicates that cumulative exposure to key factors result in fatigue failure over time which may represent an important etiological developmental factor associated with shoulder pain [24, 26]. Cumulative exposure over time may account for the findings observed in Gen2 as the younger population simply has not been exposed to the physical demands of their roles for long enough to develop shoulder pain. The cross-sectional nature of this analyses reduces the ability to infer causation however, key factors could predict shoulder pain in a working adult population.

The IPAQ is a validated self-reporting instrument for scrutinising physical activity and inactivity [27]. Participants physical activity was assessed over a 7-day period in two general areas either low, moderate, or vigorous walking-based activities or the number of hours spent sitting. While it is widely recognized that increased physical activity is essential for promoting good health [28], lowering the risk of disease development [29], and reducing premature mortality [30], our findings indicate that physical activity does not show a significant association with shoulder pain. Unfortunately, the physical activity reported did not represent any occupational specific demands performed in the 7-day period. It has been reported that single question measures such as the IPAQ can significant under-report sedentary time in comparison to selected device measures such as accelerometers, inclinometers, and pedometers [31]. It is conceivable that the lack of association between physical activity and shoulder pain identified in this study may be contributed to the sensitivity of the questionnaire employed. It has been identified that reporting accuracy is enhanced by implementing multi-item questionnaires, logs/diaries in comparison to single item questions [31]. Questionnaires intended to investigate the type and frequency of upper extremity activity could possibly identify an association between physical activity and shoulder pain.

The association of depression and anxiety with shoulder pain was investigated in the fourth objective. This produced the clearest association with the younger generation being almost twice, and the older population being more than twice as likely to report shoulder pain when also being classified has having depression or anxiety. This is in line with previous research demonstrating that patients with shoulder pain were more likely to report depression and anxiety when compared with a healthy cohort [6]. Our findings add to the existing knowledge by demonstrating this association across multiple generations in a population based sample. Though the direction of this association is not established, a bi-directional relationship has been demonstrated to exist between general pain and depression [16]. It is possible that those suffering from psychological distress are increasingly reporting shoulder pain and visa versa. A longitudinal analyses would need to be performed to further establish the strength of the relationship.

### Strengths and limitations

The samples are community-dwelling and predominantly Caucasian (85%), but are considered to be representative of the Western Australians of the same age [18]. The representativeness of Gen1 mothers compared to the Western Australian population has been investigated at six time points, including the Gen1-26 year follow-up. There were only small differences between the Gen1 mothers and the 2016 Western Australian Population Census data of women 55–64 years of age [19]. This strengthens the external validity of the results. The Gen1 sample has an age range that crosses retirement age, allowing for group comparisons of pre and post retirement age.

We recognise that this study has several key limitations. The Gen1 cohort may not entirely represent the general population of females as the participants were all mothers. Shoulder pain was self-reported with no anatomical definition or body diagram provided to the participants. This was because the question had been asked through the Orebro questionnaire. We cannot be certain whether this would have resulted in an under or overestimation of shoulder pain prevalence. Furthermore, there were not data that could be linked directly to the shoulder pain question regarding duration of pain, intensity of pain, disability, and number of episodes. This could again result in participants reporting short-lived pain that may or not be disabling in nature and potentially inflating the rates of reported shoulder pain.

We acknowledge a limitation in the collection of pain data, where theGen1 cohort were asked to report shoulder pain experienced in the last month, with Gen2 asked to report if they were currently experiencing shoulder pain. While there is evidence to indicate that recollection of pain may be accurate when reported over a one month period [32], we are unable to make direct comparisons between generations. The relationships between predictor variables and prevalence estimates could be influenced by the different recollection periods between the generations.

Our study included an extensive list of potential predictor variables, but we acknowledge that many other potential confounders may influence shoulder pain estimates. Future studies could investigate the association with co-morbidities, sleep duration, and pain at more than one site. Finally, the small number of Gen1 participants in the over 65 age group limited the power of this analysis and resulted in wide confidence intervals for the odds ratios.

## Conclusion

Overall, one month and point prevalence estimates were produced for self-reported shoulder pain in two generations of adults. Furthermore, cross-sectional associations were established between some occupational factors in older adults and depression in all adults, and shoulder pain. These results cannot determine if this relationship is causal, bi-directional or a separate common origin.

## Data Availability

The datasets analysed during the current study are not publicly available due to this not being approved by the respective ethics boards, but are available from the corresponding author on reasonable request.
